# Usefulness of ^123^I-BMIPP and ^201^TlCl nuclide scintigraphy in evaluation of myocarditis in patients with polymyositis or dermatomyositis

**DOI:** 10.1097/MD.0000000000027173

**Published:** 2021-09-10

**Authors:** Yukinori Okada, Yukiko Takakuwa, Seido Ooka, Yukihisa Ogawa, Kumito Kawahata, Yasuyuki Kobayashi, Keiichiro Yamaguchi, Yoshihiro Akashi

**Affiliations:** a St. Marianna University School of Medicine, Department of Radiology, Kawasaki, Japan; b St. Marianna University School of Medicine, Department of Internal Medicine, Division of Rheumatology and Allergy, Kawasaki, Japan; c St. Marianna University School of Medicine, Department of Internal Medicine, Division of Cardiology, Kawasaki, Japan; d Department of Proton Therapy and Tumor Imaging, St. Marianna University School of Medicine, Kawasaki, Japan.

**Keywords:** ^123^I-BMIPP/^201^TlCl scintigraphy, dermatomyositis, mismatch, polymyositis

## Abstract

To investigate the usefulness of ^123^I-BMIPP/^201^TlCl scintigraphy for evaluating the presence of myocarditis in patients with polymyositis (PM) or dermatomyositis (DM).

We performed a retrospective study of 26 patients diagnosed with new-onset active PM/DM who underwent ^123^I-BMIPP/^201^TlCl scintigraphy between 01 April 2010 and 20 March 2015. We determined the ^123^I-BMIPP/^201^TlCl ratio and grouped the patients according to presence or absence of a mismatch. We evaluated the relationship between mismatch and the laboratory and echocardiographic findings.

Mismatch was found in 13 (50%) patients. There was no statistically significant difference in age, cardiac troponin T, myoglobin, myosin light chain, aldolase levels, E wave/A wave ratio, right ventricular systolic pressure between the mismatch and non-mismatch groups. Left ventricular end-diastolic and end-systolic dimensions were significantly greater in the mismatch group (45.0 vs 42.5 mm, *P* =  < .01 and 29.5 mm vs 25.0 mm, *P* < .01). Left ventricular ejection fraction was significantly lower in the mismatch group (63.5% vs 71.5%, *P* = .04). Significant inverse correlation (r = −0.44, *P* = .03) was observed between left ventricular ejection fraction and mismatch ratio.

The use of ^123^I-BMIPP/ ^201^TlCl scintigraphy may be considered for evaluating myocarditis in patients with PM/DM.

## 1 Introduction

Polymyositis (PM) and dermatomyositis (DM) are inflammatory autoimmune disease.^[1]^ Involvement of skeletal and smooth muscles occurs throughout the body causing myopathies.^[1]^ Cardiac involvement is an important risk factor for death in patients with PM/DM due to increased risk of cardiovascular events such as myocardial infarction.^[2–4]^ The incidence of cardiac involvement has been reported to be between 9% and 72%.^[5]^ Therefore, evaluation of myocardial function in patients with PM/DM is important.

In PM/DM, myocardial function is assessed by echocardiography, which evaluates for cardiac abnormality; more specifically for left ventricular and right ventricular systolic dysfunctions that have been reported in new onset PM/DM patients.^[6]^ Left ventricular diastolic dysfunction can also occur in patients with cardiac involvement.^[7]^ Echocardiography is used for serial monitoring of global and regional left ventricular function after ischemia,but, there are limitation for ischemic memory.^[8]^

Myocardial damage from past ischemic events (ischemic memory) can be evaluated using nuclear cardiac imaging that incorporates ^123^I-BMIPP (which reflects fatty acid metabolism) and ^201^TlCl (which reflects myocardial perfusion).^[9]^^123^I-BMIPP accumulates in ischemic areas are few, with delayed recovery.^[8]^ Therefore, ^123^I-BMIPP single photon emission computed tomography will identify ischemic areas as low accumulation (defect) area and can be used to evaluate ischemic memory.^[8]^ In contrast, in the ischemic environment created by vascular spasm, coronary blood flow is preserved and ^201^TlCl accumulation does not change. The utility of ^123^I-BMIPP/^201^TlCl mismatch for the diagnosis of coronary artery disease and as a predictor of cardiac events.^[10]^ And the utility of ^123^I-BMIPP/^201^TlCl scintigraphy was reported in patients with dilated cardiomyopathy.^[11]^ Despite the increased risk of myocardial damage in patients with PM/DM, there are few reports on the use of myocardial scintigraphy to identify ischemic memory in this group of patients. We hypothesize that a mismatch between ^123^I-BMIPP and ^201^TlCl uptake will be observed in patients with PM/DM. We therefore undertook a retrospective study to evaluate the hypothesis that a mismatch between ^123^I-BMIPP and ^201^TlCl uptake is seen in PM/DM, and to evaluate its usefulness in detecting myocarditis in these patients.

## 2 Material and methods

### 2.1 Patients

We checked the collagen disease patients who received the double-nuclide myocardial scintigraphy with ^123^I-BMIPP and ^201^TlCl between April 2010 and March 2015. And we selected a total of 26 consecutive patients with PM or DM who had undergone double-nuclide myocardial scintigraphy with ^123^I-BMIPP and ^201^TlCl between April 2010 and March 2015. In all cases, the diagnosis was new-onset active PM or DM, and had not received treatment prior to the study period.

### 2.2 Scintigraphy

**Figure 1:**
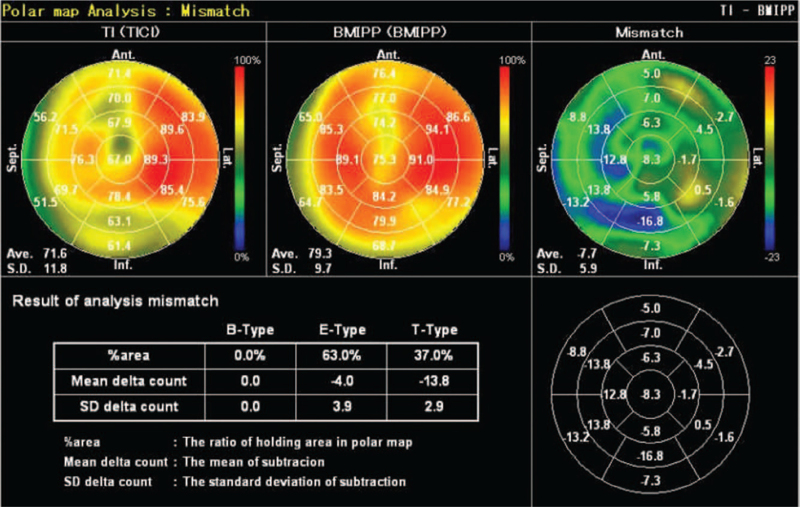
^123^I-BMIPP/^201^TlCl scintigraphy images reveal no mismatch in ^123^I-BMIPP and ^201^TlCl uptake.

**Figure 2:**
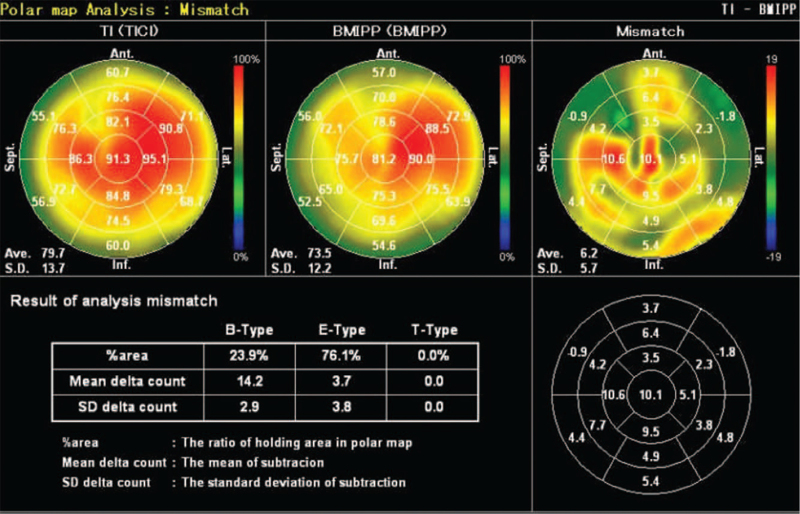
^123^I-BMIPP/^201^TlCl scintigraphy images reveal mismatch in ^123^I-BMIPP and ^201^TlCl uptake.

All patients underwent ^123^I-BMIPP/^201^TlCl myocardial scintigraphy. Intravenous administration of 111 to 148 MBq of ^123^I-BMIPP was followed by 74 MBq of ^201^TlCl. Accumulation of the 2 tracers was measured simultaneously during imaging: ^123^I-BMIPP with an ECAM (Canon Medical Systems, Otawara, Japan; imaging speed, 18 cm/min; matrix, 256 × 1024) scintillation camera, and ^201^TlCl with a GXA-7200 (Canon Medical Systems; imaging speed, 17.5 cm/min; matrix, 256 × 1024) scintillation camera. Risk estimation software (Heart Risk View, Nihon Medi-Physics Co. Ltd, Tokyo, Japan; AZE Co. Ltd, Tokyo, Japan) was then used to quantify the radioactivity and degree of mismatch. Mismatch was reported as the ratio of ^123^I-BMIPP to ^201^TlCl. Patients were grouped according to the presence or absence of mismatch. Representative images of absence and presence of mismatch are shown in Figures 1 and 2.

### 2.3 Echocardiography

Of the 26 patients, 24 underwent echocardiography, and scintigraphy was performed at a median of 4.5 days (range, 1–30 days) later. The information collected include left ventricular end-diastolic dimension, left ventricular end-systolic dimension, left ventricular ejection fraction, E wave (early diastole)/A wave (peak velocity flow in late diastole caused by atrial contraction) ratio, and right ventricular systolic pressure.

### 2.4 Laboratory tests

The laboratory tests of serum samples and the median interval between scintigraphy and each test were as follows: cardiac troponin T assay (n = 19), 7 days (range, 2–21); myoglobin assay (n = 17), 7 days (range, 0–20); myosin light-chain assay (n = 23), 7 days (range, 1–30); aldolase assay (n = 21), 7 days (range, 0–24).

### 2.5 Statistical analysis

Continuous data were presented as the mean ± SD or median values. Between-group differences were analyzed by Mann–Whitney *U* test. The relation between left ventricular ejection fraction and mismatch ratio was determined by Spearman rank order correlation coefficient. All statistical analyses were performed with EZR software, which was developed by Jichi Medical University Saitama Medical Center (Ohmiya Medical Center) as an evolution of the R statistical software (R Foundation for Statistical Computing, version 2.13.0). A *P* value (2 tail) of .05 was considered to be significant.

### 2.6 Ethical considerations

This study was conducted with the approval of the Ethics Committee of St. Marianna University School of Medicine (2976) and used opt out.

## 3 Results

### 3.1 Patient characteristics

The patient group comprised 23 women and 3 men aged 53.3 ± 15.3 years (Table 1). None of the patients had a history of ischemic heart disease, congenital heart disease, valvular heart disease, chronic renal failure, sarcoidosis, or amyloidosis. Four patients had a history of asymptomatic hypertension. All patients satisfied the diagnostic criteria by Ministry of Health 2015 about the PM/DM. There were no patients with special antibody. There were no patients with specific outcomes.

**Table 1:** Patient characteristics.

	n	*P* value
Age (yrs)		53.3 ± 15.3
Sex (m/f)	3/23	
Scintigraphy mismatch (%)	26	2.6 ± 5.9
End-diastolic dimension (mm)	24	43.9 ± 3.5
End-systolic dimension (mm)	24	27.3 ± 3.2
Ejection fraction (%)	24	67.8 ± 5.7
E/A	23	0.9 ± 0.4
DCT	22	210 ± 56.4
Right ventricular systolic pressure (mm Hg)	23	26.2 ± 7.4
Troponin T (ng/mL)	19	0.09 ± 0.11
Myoglobin (ng/mL)	17	469.7 ± 595.4
Myosin light chain (ng/mL)	23	46 ± 66.6
Aldolase (IU/L)	21	23.1 ± 21.5

### 3.2 Scintigraphy

Uptake mismatch was observed in 13 (50%) patients. The mismatch ratio was 2.6 ± 5.9%. Upon visual inspection of the images, the distributions of ^123^I-BMIPP and ^201^TlCl appeared not to be segmental or based on coronary artery segmentation.

### 3.3 Echocardiographic findings

The mean left ventricular end-diastolic dimension was 43.9 ± 3.5 mm, end-systolic dimension was 27.3 ± 3.2 mm, and ejection fraction was 67.8 ± 5.7%. The E/A ratio was 0.9 ± 0.4, and right ventricular systolic pressure was 26.2 ± 7.3 mm Hg. The mean deceleration time (DCT) was 210 ± 56.4 ms.

### 3.4 Laboratory findings

The mean values of the laboratory tests and the number of patients showing above-normal values were as follows: cardiac troponin T concentration = 0.09 ± 0.11 ng/mL (≥0.10 ng/mL, n = 5); myoglobin concentration = 469.7 ± 595.4 ng/mL (>58 ng/mL, n = 12); myosin light-chain concentration = 46.0 ± 66.6 ng/mL (≥2.5 ng/mL, n = 22); aldolase concentration = 22.3 ± 20.9 IU/L (>6 IU/L, n = 17).

### 3.5 Results of ^123^I-BMIPP and ^201^TlCl

**Figure 3:**
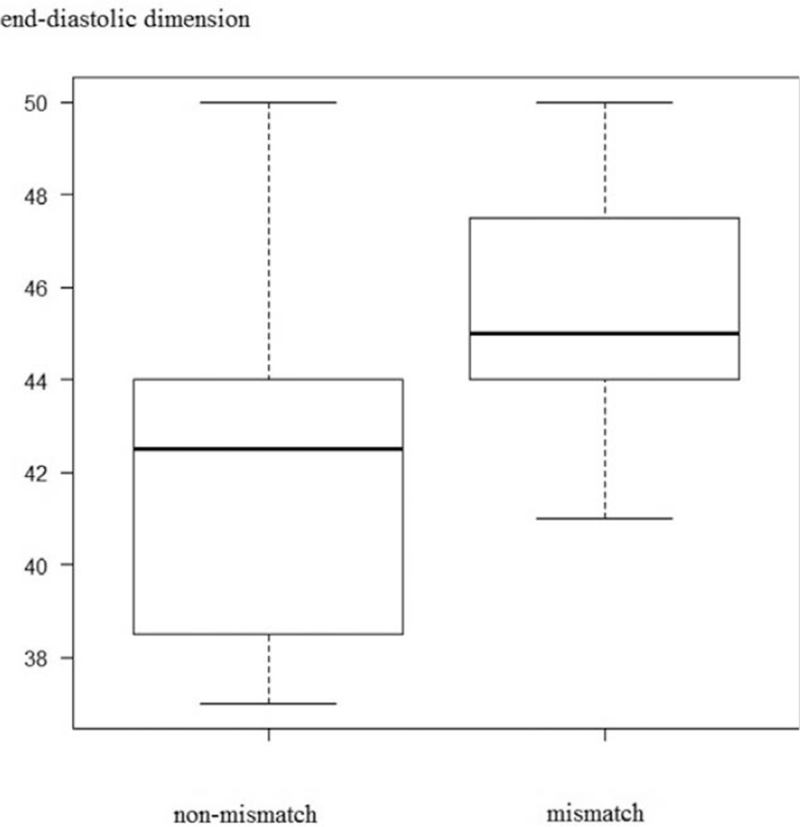
Cardiac end-diastolic dimensions in the non-mismatch and mismatch groups.

**Figure 4:**
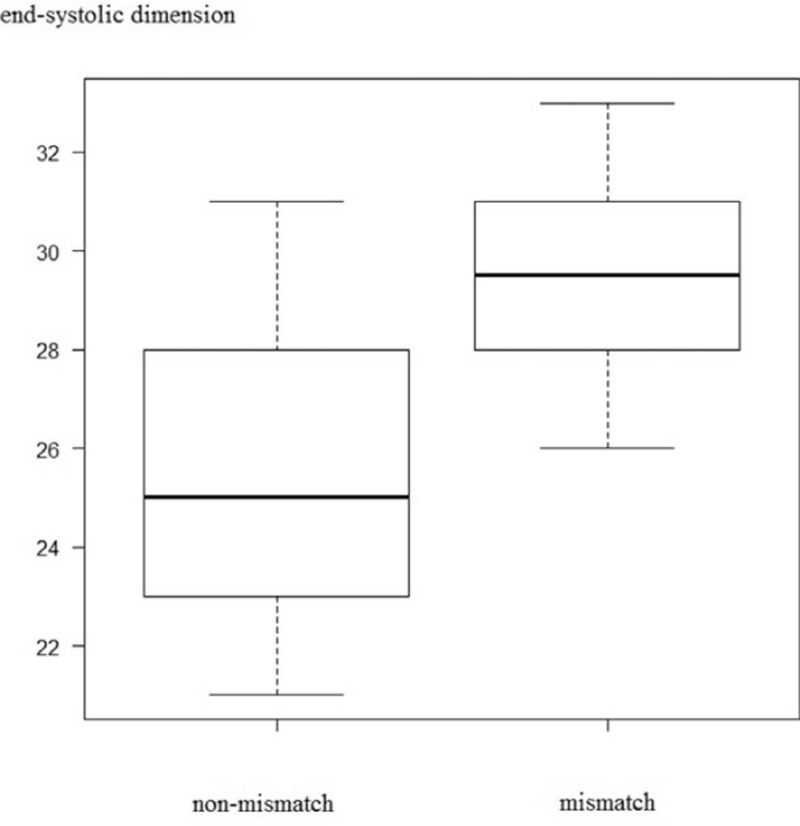
Cardiac end-systolic dimensions in the non-mismatch and mismatch groups.

**Figure 5:**
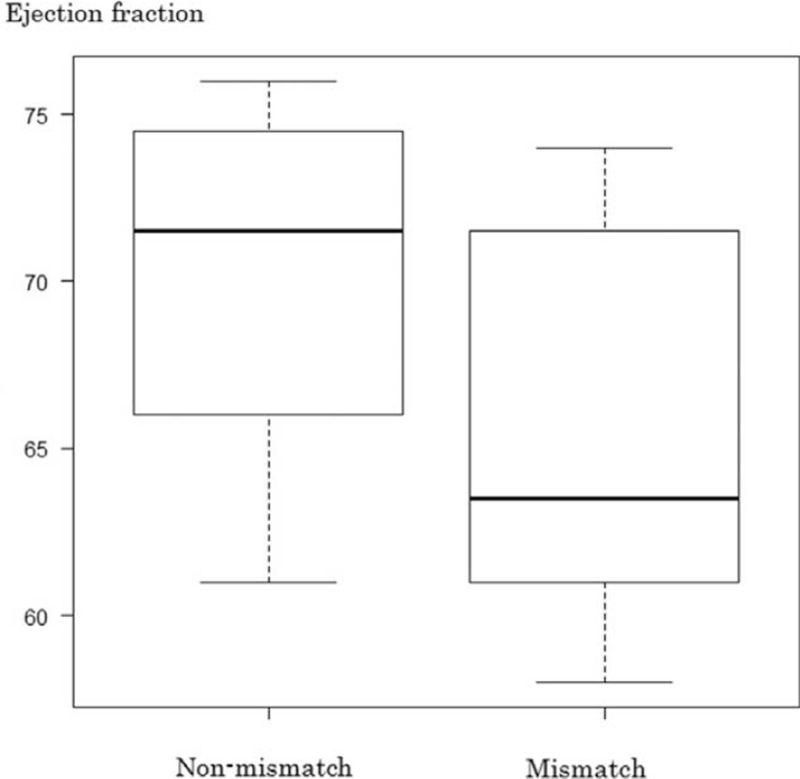
Cardiac ejection fraction in the non-mismatch and mismatch groups.

**Figure 6:**
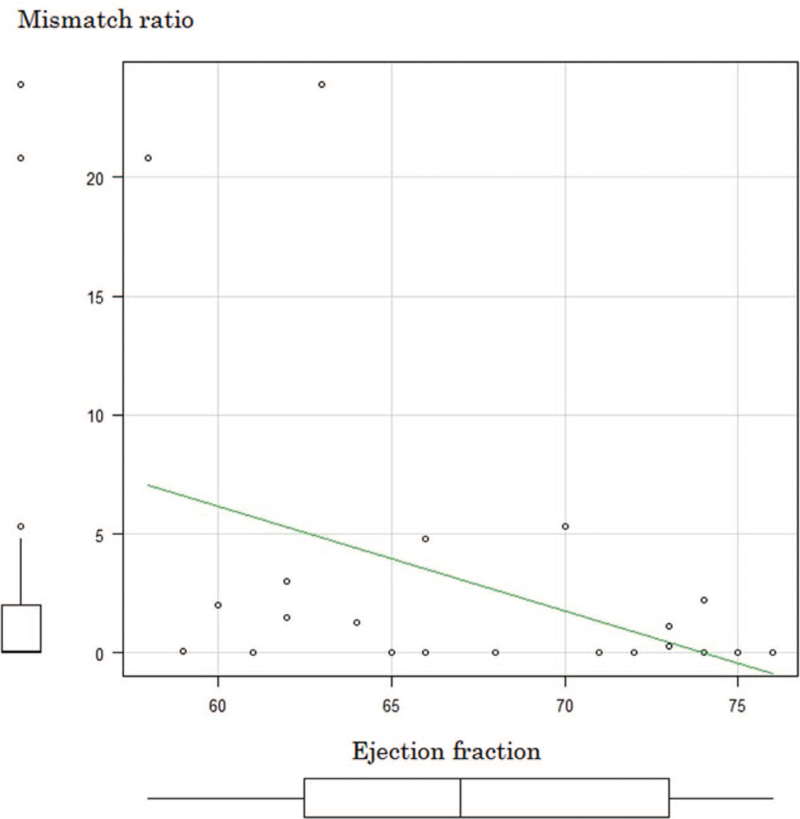
Scatterplot and regression line showing the correlation between left ventricular ejection fraction and mismatch ratio.

The median mismatch ratio was 0.05% (range, 0%–23.9%). The left ventricular end-diastolic and end-systolic dimensions were significantly greater in the mismatch group (45.0 mm vs 42.5 mm, *P* = .01 and 29.5 mm vs 25.0 mm; *P* < .01) (Figs. 3 and 4). The left ventricular ejection fraction was significantly lower in the mismatch group (63.5% vs 71.5%; *P* = .04) (Fig. 5). Significant inverse correlation (r = −0.44; *P* = .03) was found between left ventricular ejection fraction and the mismatch ratio (Fig. 6). The results of the ^123^I-BMIPP and ^201^TlCl mismatch analysis are shown in Table 2. No statistically significant difference was found between the mismatch and non-mismatch groups in terms of age, cardiac troponin T, myoglobin, myosin light chain, aldolase levels, E/A ratio, right ventricular systolic pressure or DCT.

**Table 2:** Results of ^123^I-BMIPP/^201^TlCl mismatch analysis.

	n	Non-mismatch group (n = 13)	Mismatch group (n = 13)	*P* value
Age (yrs)	26	58	51	n.s.
Scintigraphic mismatch ratio (%)	26	0	2.00	<.01
End-diastolic dimension (mm)	24	42.5	45.0	.01
End-systolic dimension (mm)	24	25.0	29.5	<.01
Ejection fraction (%)	24	71.5	63.5	.04
E/A	23	0.75	0.77	n.s.
DCT	22	198	204	n.s
Right ventricular systolic pressure (mm Hg)	23	24.3	25.1	n.s.
Troponin T (ng/mL)	19	0.09	0.11	n.s.
Myoglobin (ng/mL)	17	159.0	437.5	n.s.
Myosin light chain (ng/mL)	23	27.8	9.10	n.s.
Aldolase (IU/L)	21	16.0	10.1	n.s.

## 4 Discussion

There are some reports about the utility of ^123^I-BMIPP/^201^TlCl mismatch in the diagnosis of myocarditis. The mismatch between ^123^I-BMIPP and ^201^TlCl uptake is useful for diagnosing active cardiac sarcoidosis.^[12]^

In this study, ^123^I-BMIPP/^201^TlCl mismatch was observed in 50% (13/26) of the patients, the left ventricular end-diastolic and end-systolic dimensions were significantly greater in the mismatch group, and the left ventricular ejection fraction was significantly lower in the mismatch group. The mean left ventricular end-diastolic dimension of 43.9 ± 3.5 mm, end-systolic dimension of 27.3 ± 3.2 mm, and ejection fraction of 67.8 ± 5.7% are similar to that of previous reports (ventricular end-diastolic dimension/end-systolic dimensions/ejection fraction of 48.0 ± 3.82 mm/31.25 ± 3.07 mm/64.2 ± 4.76% and 47.0 ± 1.0 mm/2.83 ± 0.8 mm/60.8 ± 0.8%.^[6,7]^ Five patients (19%) in our study showed abnormal cardiac troponin T level, which is lower than that in a previous report (41%).^[13]^ Elevated cardiac troponin T level suggests cardiac ischemic damage and ischemic memory, which reduces cardiac function. However, the cardiac damages was small, accounting for the echocardiography and cardiac troponin T change.

The cardiac involvement in PM/DM-myocradial inflammation, degenerative change, necrosis. 46.4% patients died by heart disease in PM/DM.^[5]^ So the cardiac damage evaluation is important. In this study, we used ^123^I-BMIPP/^201^TlCl scintigraphy as a screening, there are some abnormal value about cardiac troponin T, myoglobin, myosin light chain. But, our results should be interpreted in light of the limitations of our study, which include its retrospective design and the small number of patients, Further, we did not evaluate for myocardial function using cardiac magnetic resonance imaging. Cardiac magnetic resonance imaging is useful, but, there are side effect-especially, allergy, kidney damage. Moreover, there are sudden change risk by the load. One hand, ^123^I-BMIPP/^201^TlCl scintigraphy do not need the motion or drug load. 2D echocardiography is useful, but there are skill variation between the physician. Other hand, ^123^I-BMIPP/^201^TlCl scintigraphy do not depend on the physician skill and have the high quality of objectivity. So we used the ^123^I-BMIPP/^201^TlCl scintigraphy. But we did not identify the mismatch site. Additional research is needed in this regard. From these results, the cardiac damage evaluation is necessary.

In conclusion, we found that mismatch between ^123^I-BMIPP and ^201^TlCl uptake was useful for early detection of PM/DM-associated cardiomyopathy. A prospective clinical trial involving a large number of patients is needed to confirm the mismatch phenomenon in patients with PM/DM.

## Author contributions

**Data curation:** Yukinori Okada, Yukiko Takakuwa.

**Data analysis:** Yukinori Okada, Yukiko Takakuwa.

**Formal and result check:** Seido Ooka, Yukihisa Ogawa.

**Initial idea:** Yukinori Okada, Yukiko Takakuwa.

**Project administration:** Yasuyuki Kobayashi, Keiichiro Yamaguchi, Yoshihiro Akashi, Kimito Kawahata.
